# Flexural Performance of CLT Plates Under Coupling Effect of Load and Moisture Content

**DOI:** 10.3390/ma18245597

**Published:** 2025-12-12

**Authors:** Jinpeng Xu, Tianyi Zhang, Huanyu Wang, Aiguo Zhao, Peng Wu

**Affiliations:** College of Civil Engineering, Nanjing Tech University, Nanjing 211816, China; xujinpeng@njtech.edu.cn (J.X.); zhangtianyi@njtech.edu.cn (T.Z.); wanghuany@njtech.edu.cn (H.W.); shiang_37@163.com (A.Z.)

**Keywords:** CLT plate, moisture-dependent property, load–moisture coupling, state-space equation, transfer matrix method

## Abstract

As a green-material structure, cross-laminated timber (CLT) has attracted increasing attention and applications in construction. This study presents an analytical model for a CLT plate under the coupling effect of load and moisture content, where the moisture-induced deformation and moisture-dependent properties are both considered. In the analytical model, state-space equations for moisture variables and for stresses and displacements in the CLT plate are established based on moisture diffusion theory and three-dimensional elasticity theory, respectively. Using the transfer matrix method, the relationships of moisture variables, stresses, and displacements between any two layers of the CLT plates are formulated. The analytical solutions are then determined by the load and moisture conditions applied to the top and bottom surfaces. Comparative analysis indicates that the proposed solution surpasses finite element methods in both computational accuracy and efficiency. In addition, the stress and displacement patterns of CLT plates under pure load and pure moisture conditions, as well as their interrelations, are investigated through a decoupled analysis. An applicable modified superposition principle is then proposed. Finally, a detailed parametric study is conducted to examine the effects of moisture distribution and wood species.

## 1. Introduction

Wood is a natural, renewable, bio-based material with a high strength-to-weight ratio, good workability, and favorable environmental performance, making it widely used in construction, furniture, and structural engineering. Cross-laminated timber (CLT) is an engineered structural wood product composed of multiple layers of sawn lumber boards arranged orthogonally and bonded with structural adhesives under pressure. The cross-laminated configuration enhances strength, stiffness, and dimensional stability in both in-plane directions, enabling CLT plates to serve as load-bearing components such as floors, walls, and roofs in modern mass timber buildings. However, CLT is highly sensitive to moisture variation; moisture exchange with the environment can induce swelling or shrinkage, resulting in deformation and stress redistribution [1]. Moreover, its mechanical properties are moisture-dependent, as both stiffness and strength vary with moisture content (MC) [2]. Therefore, investigating the flexural performance of CLT plates’ load–moisture (LM) coupling environment is of great importance.

The mechanical behavior of CLT plates is primarily studied through experimental testing. These methods enable direct observation of deformation, failure modes, and stress redistribution under realistic load and boundary conditions. As such, experimental testing provides reliable benchmark data, forming a crucial foundation for model validation, material characterization, and the design optimization of CLT structures. He et al. [3] studied the flexural, shear, and compressive properties of three-layer vs. five-layer CLT plates made of black spruce and found that increasing thickness alters flexural strength and stiffness behavior. Nakagawa et al. [4] examined sugi CLT composed of different lamina thicknesses and reported the effects on flexural modulus and failure behavior. Olsson et al. [5] conducted dynamic and quasi-static measurements of the longitudinal modulus of elasticity and effective rolling shear modulus for CLT plates made of Norway spruce and Scots pine. Altaher Omer Ahmed et al. [6] investigated hybrid CLT beams with hardwood faces and softwood cores, observing enhanced flexural strength compared to conventional plates. Kawaai et al. [7] measured the out-of-plane shear strength of Japanese larch CLT under different layups and spans. Gülzow et al. [8] reported that flexural and shear stiffness decrease as moisture content increases. Huang [9] evaluated creep behavior under cyclic moisture changes, illustrating coupling between load and moisture. Zhou et al. [10] studied in-plane shear failure modes of CLT plates and analyzed crack morphology under load configurations. Yamagata et al. [11] used asymmetric four-point bending to assess in-plane shear modulus variations. Kurzinski and Crovella [12] demonstrated that angle-ply lamination improves the out-of-plane flexural stiffness of CLT plates. Takanashi et al. [13] evaluated the long-term flexural properties of Japanese larch CLT. Li et al. [14] assessed rolling shear strength using short-span bending plus modified planar shear tests.

In addition to experimental methods, analytical models are crucial for understanding the mechanical behavior of CLT plates. They are cost-effective, time-efficient, and versatile, allowing for the exploration of various load conditions and material variations without the need for extensive testing. Analytical models also help predict performance and optimize designs, providing valuable insights when combined with experimental data, which ultimately enhance structural assessments and design guidelines. For instance, Dobeš et al. [15] conducted both analytical and finite element simulations to compare the flexural stiffness and deformation of 3-, 5-, and 7-layer Nordic spruce CLT plates, demonstrating strong agreement between laboratory tests, classical analytical methods, and finite element results. Olsson et al. [5] assessed the longitudinal modulus of elasticity and effective rolling shear modulus in Norway spruce and Scots pine CLT using both quasi-static and dynamic methods, establishing key stiffness parameters for modeling. Yang et al. [16] investigated ply-laminated CLT plates with varying lamina configurations, including hybrid boards, quantifying the influence of layer geometry on flexural and shear properties. Yusof et al. [17] compared the mechanical responses of CLT plates manufactured with different adhesives, linking changes in moduli to physical properties and MC absorption behavior. Silva et al. [18] analyzed strain variation in CLT plates subjected to changes in MC, finding measurable reductions in stiffness parameters as MC increased. Aloisio et al. [19] evaluated the sensitivity of flexural stiffness in adhesive-free dowelled CLT plates under varying MC conditions. Recently, Brandstätter et al. [20] developed a numerical simulation framework for moisture uptake and drying in CLT plates following water infiltration through end-grain surfaces, demonstrating that spatial MC gradients significantly influence stiffness and flexural behavior. Schmidt and Riggio [21] monitored MC performance in full-scale CLT floor and shear-wall elements during construction, providing real-world MC data for numerical models. Additionally, Fu et al. [22] investigated the effects of MC and grain direction on the compressive yield strength, modulus of elasticity, and shear modulus of beech wood. Florisson et al. [23] developed a numerical model to simulate the moisture flow and hygro-mechanical visco-elastic behavior of wood, laying the foundation for CLT modeling and moisture-dependent properties (MDPs). Collectively, these studies illustrate the progression from purely mechanical modeling to the inclusion of MDP, setting the stage for advanced hygro-mechanical coupling frameworks in CLT research.

In the studies mentioned above, most existing analytical models focus primarily on load effects, while research explicitly considering the combined impact of moisture and mechanical loads remains limited. The few studies that do incorporate moisture effects often overlook MDP and assume transverse shear deformation. Neglecting MDP and assuming transverse shear deformation in CLT flexural analysis can lead to inaccurate performance predictions. Ignoring MDP disregards moisture-induced changes in stiffness and strength, while assuming transverse shear deformation introduces errors in deflection and stress calculations. To address the research gaps, this study establishes an analytical model based on three-dimensional (3D) elasticity theory, which does not assume any form of transverse shear deformation, to study the flexural performance of CLT plate under the coupling effect of load and MC. Moreover, both moisture-induced deformation and MDP are considered. In the analytical model, based on moisture diffusion theory, a state-space equation for MC and moisture flux is established. Additionally, a state-space equation for stress and displacement is formulated using the control equations of 3D elasticity theory. Considering the load and MC conditions on the top and bottom surfaces of the CLT plate, the analytical solutions for MC, stress, and displacement are derived using the transfer matrix method. The developed model aims to (i) elucidate the load–moisture interaction mechanisms of CLT plates under various moisture contents and load conditions; (ii) investigate the influence of different wood species and their anisotropic elastic and hygroscopic properties on the redistribution of moisture-induced stresses and the resulting deformation behavior; and (iii) examine the effects of moisture gradients at the top and bottom surfaces on the symmetry and nonlinear characteristics of stress and displacement fields, thereby providing theoretical guidance for predicting and mitigating moisture-induced deformation in service environments.

## 2. Analytical Model for CLT Plates

**Figure 1:**
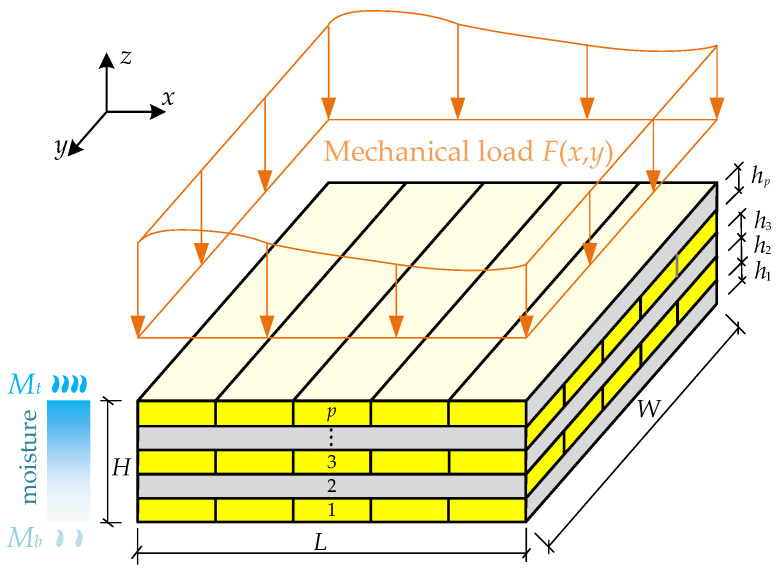
CLT plate under LM coupling condition.

As presented in Figure 1, the research object analyzed in this work is a CLT plate, consisting of *p* timber layers made of same of different woods. The CLT plate is subjected to a distributed load *F*(*x*, *y*) on its upper surface and exposed to a non-uniform moisture field with contents *M_t_* and *M_b_* at the top and bottom surfaces, respectively. In a moisture environment, both moisture deformation and the MDP of wood are considered. The plate is simply supported along four edges, and the adjacent layers are perfectly bonded by adhesives without interfacial slip. A Cartesian coordinate system *o-xyz* is established, where *L*, *W* and *H* represent the length, width, and total thickness of the CLT plate, respectively, and *h_i_* denotes the thickness of the *i*-th layer.

### 2.1. Governing Equations

For the distribution of MC within the CLT plate, based on the moisture diffusion theory [24], the moisture diffusion equation for the *i*-th layer is

(1) $$\frac{\partial^{2}M^{i}}{\partial x^{2}}+\frac{\partial^{2}M^{i}}{\partial y^{2}}+\frac{\partial^{2}M^{i}}{\partial z^{2}}=0,$$

where *M^i^* represents the MC of the *i*-th timber layer. The *z*-direction moisture flux $Q_{z}^{i}$ can be expressed by the gradient of the moisture concentration in the *z*-direction, according to Fick’s law of moisture diffusion [25], as follows:

(2) $$Q_{z}^{i}=-\lambda_{z}^{i}\frac{\partial M^{i}}{\partial z},$$

where and $\lambda_{z}^{i}$ represents the *z*-direction moisture diffusion coefficient.

For the flexural performance of the CLT plate under the LM coupling condition, the stresses and displacements are analyzed in accordance with 3D elasticity theory [26]. Considering both moisture deformation and the moisture-dependent modulus, the governing equations, including the constitutive equations, geometric equations and equilibrium equations, for the CLT plate are expressed in tensor form as follows:

(3) $$\sigma_{j}^{i}=c_{jk}^{i}(\varepsilon_{k}^{i}-\beta_{k}^{i}),$$

(4) $$\gamma_{kj}^{i}=0.5(u_{k,\;j}^{i}+u_{j,\;k}^{i}),$$

(5) $$\sigma_{kj,\;j}^{i}=0,$$

where $\sigma^{i}$, $\varepsilon^{i}$, $\beta_{k}^{i}$, $u^{i}$ and $c_{jk}^{i}$ represent the tensors of stress, strain, the moisture expansion coefficient, displacement and the stiffness coefficient of the *i*-th timber layer, which can be expressed in vector or matrix form:

[ci]=ξic11ic12ic13i000 c22ic23i000  c33i000   c44i00 sym.  c55i0     c66i,[βki]=[βxiβyiβzi000]T, [ui]=[uxiuyiuzi]T.

in which $\xi_{i}$ denotes the MDP coefficient of the wood. The stiffness coefficient $c_{jk}^{i}$ in the constitutive equation considers the orthotropic property of wood, which displays distinct mechanical properties in the longitudinal, radial, and tangential directions due to its biological and morphological characteristics. Meanwhile, $c_{jk}^{i}$ can also be expressed by the elastic modulus *E*, shear modulus *G* and Poisson’s ratio *μ*, as follows

$$c_{11}^{i}=\frac{1-\mu_{23}^{i}\mu_{32}^{i}}{E_{2}^{i}E_{3}^{i}\Delta^{i}},\;c_{12}^{i}=\frac{\mu_{12}^{i}+\mu_{13}^{i}\mu_{32}^{i}}{E_{1}^{i}E_{3}^{i}\Delta^{i}},\;c_{13}^{i}=\frac{\mu_{13}^{i}+\mu_{12}^{i}\mu_{23}^{i}}{E_{1}^{i}E_{2}^{i}\Delta^{i}},\;c_{22}^{i}=\frac{1-\mu_{13}^{i}\mu_{31}^{i}}{E_{1}^{i}E_{3}^{i}\Delta^{i}}$$

$$c_{23}^{i}=\frac{\mu_{23}^{i}+\mu_{21}^{i}\mu_{13}^{i}}{E_{1}^{i}E_{2}^{i}\Delta^{i}},\;c_{33}^{i}=\frac{1-\mu_{12}^{i}\mu_{21}^{i}}{E_{1}^{i}E_{2}^{i}\Delta^{i}},\;c_{44}^{i}=G_{23}^{i},\;c_{55}^{i}=G_{13}^{i},\;c_{66}^{i}=G_{12}^{i},$$

(6) $$\Delta^{i}=\left|\begin{array}{ccc} \frac{1}{E_{1}^{i}} & -\frac{\mu_{21}^{i}}{E_{2}^{i}} & -\frac{\mu_{31}^{i}}{E_{3}^{i}}\\ -\frac{\mu_{12}^{i}}{E_{1}^{i}} & \frac{1}{E_{2}^{i}} & -\frac{\mu_{32}^{i}}{E_{3}^{i}}\\ -\frac{\mu_{13}^{i}}{E_{1}^{i}} & -\frac{\mu_{23}^{i}}{E_{2}^{i}} & \frac{1}{E_{3}^{i}} \end{array}\right|,$$

in which the subscripts 1, 2, and 3 denote the principal material directions corresponding to the global *x*-, *y*-, and *z*-axes, respectively. Meanwhile, 1, 2, and 3 are associated with the longitudinal, radial, and tangential directions of the wood, abbreviated as the *L*, *R*, and *T* directions, respectively. Specifically, when the longitudinal direction of a wood lamina is aligned with the *x*-axis, the mapping is (1, 2, 3)–(*L*, *T*, *R*). Conversely, when the longitudinal direction of the lamina is perpendicular to the *x*-axis, the mapping changes to (1, 2, 3)–(*T*, *L*, *R*).

Here, a CLT plate with simply supported edges is considered, where vertical displacement is constrained at the boundaries, while free rotation is permitted. Three common representations for simply supported boundaries are typically employed: the stress boundary, displacement boundary, and stress–displacement mixed boundary. These representations produce nearly identical results, with only minor differences observed in regions close to the boundaries [26]. To facilitate the solution of the equations, the mixed boundary condition is adopted, which can be expressed as

$$\sigma_{x}^{i}=u_{y}^{i}=u_{z}^{i}=0,\;\mathrm{at}\;x=0,L,$$

(7) $$\sigma_{y}^{i}=u_{x}^{i}=u_{z}^{i}=0,\;\mathrm{at}\;y=0,W.$$

### 2.2. Solutions of Moisture, Stresses and Displacements of CLT Plate

Since the governing equations of moisture, moisture flux, stresses and displacements for the CLT plate constitute a set of partial differential equations, they are difficult to solve directly. Here, in accordance with the simply supported boundary conditions, they can be expressed in specific forms of Fourier series, as follows

(8) $$\left[\begin{array}{c} M^{i}\\ Q_{z}^{i}\\ u_{x}^{i}\\ u_{y}^{i}\\ u_{z}^{i} \end{array}\right]=\sum_{m=1}^{\infty }\sum_{n=1}^{\infty }\left[\begin{array}{c} M_{mn}^{i}(z)\sin(a_{m}x)\sin(b_{n}y)\\ Q_{z,mn}^{i}(z)\sin(a_{m}x)\sin(b_{n}y)\\ u_{x,mn}^{i}\cos(a_{m}x)\sin(b_{n}y)\\ u_{y,mn}^{i}\sin(a_{m}x)\cos(b_{n}y)\\ u_{z,mn}^{i}\sin(a_{m}x)\sin(b_{n}y) \end{array}\right],\;\left[\begin{array}{l} \sigma_{x}^{i}\\ \sigma_{y}^{i}\\ \sigma_{z}^{i}\\ \tau_{yz}^{i}\\ \tau_{xz}^{i}\\ \tau_{xy}^{i} \end{array}\right]=\sum_{m=1}^{\infty }\sum_{n=1}^{\infty }\left[\begin{array}{l} \sigma_{x,mn}^{i}\sin(a_{m}x)\sin(b_{n}y)\\ \sigma_{y,mn}^{i}\sin(a_{m}x)\sin(b_{n}y)\\ \sigma_{z,mn}^{i}\sin(a_{m}x)\sin(b_{n}y)\\ \tau_{yz,mn}^{i}\sin(a_{m}x)\cos(b_{n}y)\\ \tau_{xz,mn}^{i}\cos(a_{m}x)\sin(b_{n}y)\\ \tau_{xy,mn}^{i}\cos(a_{m}x)\cos(b_{n}y) \end{array}\right],$$

where $a_{m}=m\pi /L$, $b_{n}=n\pi /W$. Substituting the expanded expressions from Equation (8) into the governing equations of Equations (1)–(5) and applying the state space method, that is, placing the derivatives of the out-of-plane quantities with respect to *z* on the left-hand side and the remaining quantities on the right-hand side, yields the state-space equations for the out-of-plane quantities as follows:

(9) $$\frac{d}{dz}X_{mn}^{i}(z)=S_{mn}^{i}X_{mn}^{i}(z)+M_{mn}^{i}(z),$$

where $X_{mn}^{i}={\left[\begin{array}{cccccccc} M_{mn}^{i} & Q_{z,mn}^{i} & u_{x,mn}^{i} & u_{y,mn}^{i} & u_{z,mn}^{i} & \sigma_{z,mn}^{i} & \tau_{xz,mn}^{i} & \tau_{yz,mn}^{i} \end{array}\right]}^{T}$ denotes the vector comprising the eight out-of-plane quantities, and the details of $S_{mn}^{i}$ and $M_{mn}^{i}$ are given in Appendix A. According to the theory of matrix differential equations, the general solutions of Equation (9) are determined as

(10) $$X_{mn}^{i}(z)=e^{S_{mn}^{i}(z-d_{i-1})}X_{mn}^{i}(d_{i-1})+\int_{d_{i-1}}^{z}e^{S_{mn}^{i}(z-\eta )}M_{mn}^{i}(\eta )d\eta ,$$

Since the adjacent timber layers are well-bonded, the MC, the out-of-plane moisture, moisture flux, stresses and displacements are all continuous along the *z* direction at the interface:

(11) $$X_{mn}^{i}(d_{i-1})=X_{mn}^{i-1}(d_{i-1}),\;i=1,2\ldots p.$$

By successively and repeatedly applying Equations (10) and (11), the relationships of $X_{mn}^{i}$ between the *i*-th and first timber layers can be established:

$$X_{mn}^{i}(z)=\Phi_{mn}^{i}(z)X_{mn}^{1}(0)+\Omega_{mn}^{i}(z),\;\Phi_{mn}^{i}(z)=e^{S_{m}^{i}(z-d_{i-1})}\prod_{k=i-1}^{1}e^{S_{m}^{k}h_{k}}X_{mn}^{1}(0),$$

(12) $$\Omega_{mn}^{i}(z)=e^{S_{m}^{i}(z-d_{i-1})}\sum_{k=1}^{i-2}\left\{\left[\prod_{j=i-1}^{k+1}e^{S_{m}^{j}h_{j}}\right]K_{k}(h_{k})\right\}+e^{S_{m}^{i}(z-d_{i-1})}K_{i-1}(h_{i-1})+K_{i}(z).$$

where

$$K_{i}(z)=\int_{d_{i-1}}^{z}e^{S_{mn}^{i}(z-\eta )}M_{mn}^{i}(\eta )d\eta .$$

Up to this point, only $X_{mn}^{1}(0)$ in the above equation, i.e., the out-of-plane quantities at the bottom surface of the CLT plate, remains undetermined; it can be determined by the surface conditions:

$$M^{p}=M_{t},\;\sigma_{z}^{p}=-F(x,y),\;\tau_{xz}^{p}=\tau_{yz}^{p}=0,\;\mathrm{at}\;z=H.$$

(13) $$M^{1}=M_{b},\;\sigma_{z}^{1}=\tau_{xz}^{1}=\tau_{yz}^{1}=0,\;\mathrm{at}\;z=0,$$

with letting *z* = *H* and *i* = *p* in Equation (12) and substituting Equation (13) into it, we have the following:

(14) $$X_{mn}^{p}(H)=\Phi_{mn}^{p}(H)X_{mn}^{1}(0)+\Omega_{mn}^{p}(H),$$

where

$$\left[\begin{array}{c} F_{mn}\\ M_{t,mn}\\ M_{b,mn} \end{array}\right]=\frac{4}{LW}\int_{0}^{a}\int_{0}^{b}\left[\begin{array}{c} F(x,y)\\ M_{t}\\ M_{b} \end{array}\right]\sin(a_{m}x)\sin(b_{n}y)dxdy.$$

According to the above relationship, the unknowns in $X_{mn}^{1}(0)$ can be determined as

(15) $$\left[\begin{array}{c} Q_{z,mn}^{1}(0)\\ u_{x,mn}^{1}(0)\\ u_{y,mn}^{1}(0)\\ u_{z,mn}^{1}(0) \end{array}\right]={\left[\begin{array}{cccc} \Phi_{mn}^{p,12} & \Phi_{mn}^{p,13} & \Phi_{mn}^{p,14} & \Phi_{mn}^{p,15}\\ \Phi_{mn}^{p,62} & \Phi_{mn}^{p,63} & \Phi_{mn}^{p,64} & \Phi_{mn}^{p,65}\\ \Phi_{mn}^{p,72} & \Phi_{mn}^{p,73} & \Phi_{mn}^{p,74} & \Phi_{mn}^{p,75}\\ \Phi_{mn}^{p,82} & \Phi_{mn}^{p,83} & \Phi_{mn}^{p,84} & \Phi_{mn}^{p,85} \end{array}\right]}^{-1}\left(\left[\begin{array}{c} M_{t,mn}\\ -F_{mn}\\ 0\\ 0 \end{array}\right]-{\left.\left[\begin{array}{c} \Omega_{mn}^{p,1}\\ \Omega_{mn}^{p,6}\\ \Omega_{mn}^{p,7}\\ \Omega_{mn}^{p,8} \end{array}\right]\right|}_{z=H}\right.\;\left.-\left[\begin{array}{cccc} \Phi_{mn}^{p,11} & \Phi_{mn}^{p,16} & \Phi_{mn}^{p,17} & \Phi_{mn}^{p,18}\\ \Phi_{mn}^{p,61} & \Phi_{mn}^{p,66} & \Phi_{mn}^{p,67} & \Phi_{mn}^{p,68}\\ \Phi_{mn}^{p,71} & \Phi_{mn}^{p,76} & \Phi_{mn}^{p,77} & \Phi_{mn}^{p,78}\\ \Phi_{mn}^{p,81} & \Phi_{mn}^{p,86} & \Phi_{mn}^{p,87} & \Phi_{mn}^{p,88} \end{array}\right]\left[\begin{array}{c} M_{b,mn}\\ 0\\ 0\\ 0 \end{array}\right]\right),$$

By substituting $X_{mn}^{1}(0)$ back into Equation (14), the analytical solutions for the out-of-plane quantities of any layer of the CLT plate are solved. According to the governing equations, Equations (3)–(5), the in-plane quantities of the CLT plate can be expressed by the out-of-plane ones, as follows

(16) $$\left[\begin{array}{c} \sigma_{x,mn}^{i}\\ \sigma_{y,mn}^{i}\\ \tau_{xy,mn}^{i} \end{array}\right]=\left[\begin{array}{ccc} -f_{1}^{i}a_{m} & -f_{2}^{i}b_{n} & \frac{c_{13}^{i}}{c_{33}^{i}}\\ -f_{2}^{i}a_{m} & -f_{3}^{i}b_{n} & \frac{c_{23}^{i}}{c_{33}^{i}}\\ c_{66}^{i}b_{n} & c_{66}^{i}a_{m} & 0 \end{array}\right]\left[\begin{array}{c} u_{x,mn}^{i}\\ u_{y,mn}^{i}\\ \sigma_{z,mn}^{i} \end{array}\right]+\left[\begin{array}{c} -f_{1}^{i}a_{x}^{i}-f_{2}^{i}b_{y}^{i}\\ -f_{2}^{i}a_{x}^{i}-f_{3}^{i}b_{y}^{i}\\ 0 \end{array}\right]M_{mn}^{i}.$$

**Figure 2:**
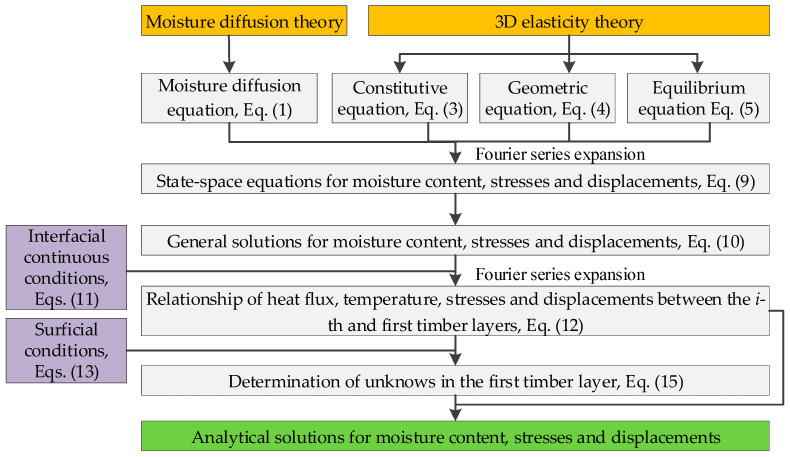
Flowchart of the present analytical model. Note: arrows indicate the sequence of derivations, yellow boxes represent the fundamental theories, gray boxes denote the derivation steps, purple boxes mark the known conditions, and green boxes indicate the final results.

To improve the readability of the analytical model, Figure 2 presents the flowchart of the proposed analytical framework.

## 3. Numerical Model

For comparison purposes, a finite element (FE) model for use in ANSYS (version: 2023) is established in this section. To ensure a fair comparison, the material properties, applied loads, moisture conditions, boundary conditions, and interfacial conditions in the FE model are kept fully consistent with those in the present analytical model. Since both MC and temperature follow the diffusion law, the thermal module is used here to analyze the moisture field of the CLT panel.

In the FE analysis, the moisture field is first obtained, after which the MDP of the wood is calculated based on Equation (17) and Table 1 using a linear elastic orthotropic material model, and the resulting moisture field, together with the external load, is then applied to the CLT panel in the structural module to determine the corresponding stresses and displacements. The FE model uses the SOLID-70 element to compute the moisture distribution and the SOLID-185 element to determine the stresses, strains, and displacements. To investigate the mesh sensitivity of the finite element model, each layer of the CLT plate is discretized into *N* elements through the thickness, while the length and width directions are uniformly divided into 100 *N* elements each, where *N* is a variable. The adjacent layers in the CLT plate are perfectly bonded, meaning that no slip or debonding occurs, which is achieved through the glue operation during modeling. The boundary conditions in the FE model are set as *u_y_* = *u_z_* = 0, at *x* = 0 and *L*; *u_x_* = *u_z_* = 0, at *y* = 0 and *W*.

## 4. Results and Discussion

In this section, the accuracy of the proposed model is verified by comparing its results with those of the FE simulations. Subsequently, the flexural response of the CLT plate subjected to the LM coupling condition is investigated by a parameter study. Unless otherwise specified, a five-layer CLT plate with dimensions of *L* = *W* = 3000 mm and *h_i_* = 30 mm serves as the reference configuration. The material parameters of the constituent wood layers are given in Table 1 [27]. According to ref. [27], the MDP coefficient with respect to MC measured at 21 °C is given by

(17) $$\xi ={\left(P_{12}/P_{g}\right)}^{\frac{0.12-M}{M_{P}-0.12}},$$

where *P*_12_ represents any elastic and shear moduli of wood at 12% MC, *P_g_* represents those of green wood, and *M_p_* is defined as the MC at which the green wood strength line intersects the logarithmic strength–MC relationship of dry wood (green).

### 4.1. Comparison and Decoupling Analyses

In this section, the present analytical solution is compared with the FE simulation, and decoupled analyses of the CLT plate under the LM-coupled environment are performed. Four conditions, including pure loads (PLs), PLs with MDP, pure moisture (PM) and the LM condition, are defined for the decoupling analysis. The research object is a CLT plate made of Spruce Sitka under the four conditions, respectively, with *F*(*x*,*y*) = 0.0001 N/mm^2^, *M_t_* = 0.205 and *M_b_* = 0.

**Figure 3:**
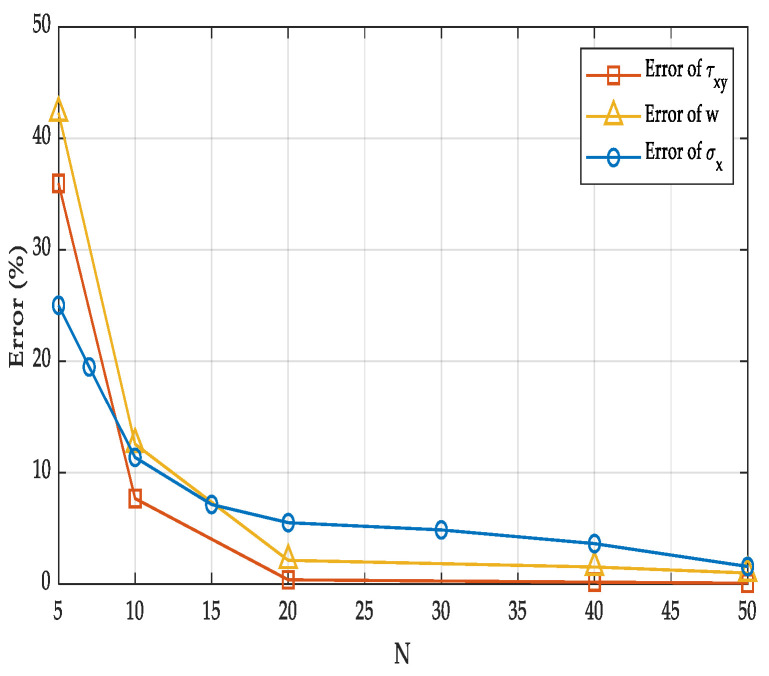
Error of the FE solution under different mesh densities.

Figure 3 shows the variations in errors of FE results for $\sigma_{x}^{i}$, $\tau_{xy}^{i}$ and $w^{i}$ with respect to *N*. As can be found from Figure 3, the error in the FE results of $\sigma_{x}^{i}$, $\tau_{xy}^{i}$ and $w^{i}$ decrease gradually as the mesh becomes finer, and the reduction is particularly rapid when 5 < *N* < 20. When *N* = 50, the errors of $\sigma_{x}^{i}$, $\tau_{xy}^{i}$ and $w^{i}$ are 1.59%, 0.0901%, and 1.02%, respectively. Therefore, in the following calculations, the mesh density in the finite element model is set to *N* = 50, resulting in a total of 6.25 × 10^9^ elements.

**Figure 4:**
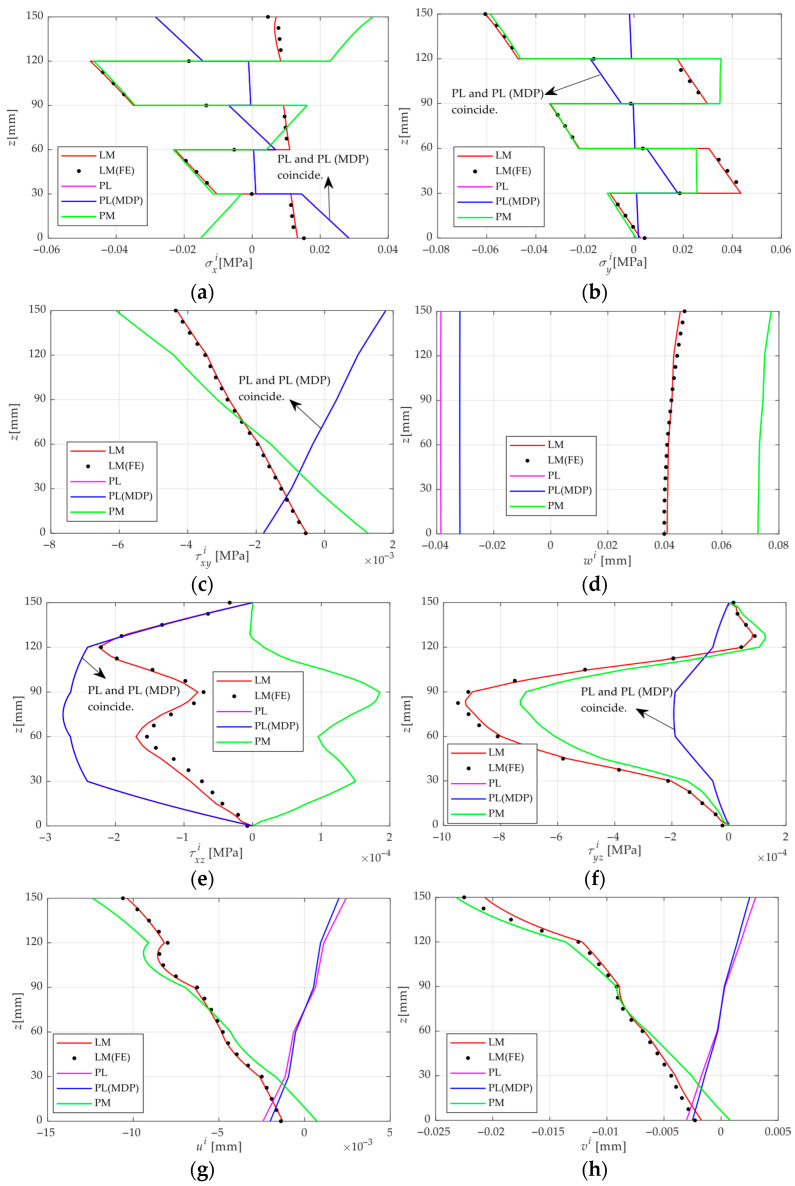
*z*-direction distributions of stresses and displacements under the four analysis environments and comparison with FE results. (**a**) $\sigma_{x}^{i}$, (**b**) $\sigma_{y}^{i}$, (**c**) $\tau_{xy}^{i}$, (**d**) *w^i^*, (**e**) $\tau_{xz}^{i}$, (**f**) $\tau_{yz}^{i}$, (**g**) *u^i^*, (**h**) *v^i^*. Note: the values of $\sigma_{x}^{i}$, $\sigma_{y}^{i}$, *w* are located at the point of *x* = 0.5 *L*, *y* = 0.5 *W*, $\tau_{xy}^{i}$, $\tau_{xz}^{i}$, $\tau_{yz}^{i}$ at *x* = 0.25 *L*, *y* = 0.25 *W*, *u* at *x* = 0, *y* = 0.5 *W* and *v* at *x* = 0.5 *L*, *y* = 0.

Figure 4 presents the through-thickness distributions of stress and displacement in the CLT plate, along with their validation against the FE analysis. It can be found from Figure 4 that (i) the through-thickness results provided by the present analytical solution agree well with those from the FE simulation overall, except the FE results show relatively large errors near the surface. While accurate FE simulations require a fine and dense mesh, the analytical solution proposed in this study achieves comparable accuracy with substantially higher computational efficiency. (ii) Under the PL condition, the external load generates flexural moments that cause the CLT plate to bend downward. In contrast, under the PM condition, the CLT plate bends upward, as the upper surface experiences higher moisture than the lower surface, leading to greater hygro-expansion. Since the flexural directions caused by the PL and PM conditions are opposite, the flexural deformation can be partially offset under the LM condition. In this special case, where moisture-induced deformation and load-induced deformation can offset each other, the bending performance of CLT plates gains additional redundancy in practical engineering applications. (iii) In the PL condition, the stress and displacement distributions of $\sigma_{x}^{i}$, $\sigma_{y}^{i}$, $\tau_{xy}^{i}$, $u^{i}$, $w^{i}$ and $v^{i}$ are antisymmetric along the thickness direction, whereas $\tau_{xz}^{i}$, $\tau_{yz}^{i}$ shows symmetry. Under PM action, no symmetry is observed, and higher stress and displacement levels occur near the upper surface, where the MC is relatively high. (iv) Under PL action, the maximum of $\sigma_{x}^{i}$, $\tau_{xy}^{i}$, $u^{i}$ and $v^{i}$ appear at the top or bottom surface of the plate, whereas $\tau_{xz}^{i}$, $\tau_{yz}^{i}$ and $\sigma_{y}^{i}$ reach their maximum values at the mid-plane. In contrast, under PM action, due to the differential moisture-induced expansion among layers, the maximum stresses are primarily concentrated at the interfaces between adjacent layers. (v) Analysis of the stress and displacement results reveals the limitations of the traditional superposition principle, i.e., PL + PM ≠ LM. This phenomenon is not significant for stresses but is more pronounced for displacements. The maximum errors of $u^{i}$, $v^{i}$ and $w^{i}$ predicted by the traditional superposition principle are 18.2%, 17.6% and 16.3%, respectively. Furthermore, when the effects of the MDP of wood are considered in the PL condition, the superposition principle is fully satisfied, i.e., PL(MDP) + PM = LM. This is referred to in this study as the modified superposition principle. This modified superposition principle is fundamentally consistent with the superposition principle for combined load and temperature effects reported in the literature [28], as both are driven by changes in the material’s mechanical properties caused by moisture content or temperature. This modified superposition principle provides guidance for the design and fabrication of CLT plates under combined moisture and mechanical loading in practical engineering, and facilitates superposed calculations for engineers.

A comparison of deflection in the CLT plate among the present analytical results, FE simulations and the existing experimental data [3] is carried out. In Ref. [3], extensive bending experiments were performed on CLT panels made of Canadian black spruce, including both three-layer and five-layer configurations. The three-layer specimens had a total length of 3300 mm and consisted of 35/35/35 mm laminations, whereas the five-layer specimens were 4800 mm long with a 35/25/35/25/35 mm layup. All panels were tested in the major strength direction. Table 2 lists the deflection of the CLT plate obtained from the present analytical model, the FE simulations and the experimental data at the elastic limit. It can be found from Table 2 that the present and FE results are close to the experimental results, and the errors of the three-layer and five-layer CLT plates between the present and experimental results are 8.14% and 9.26%, respectively.

**Figure 5:**
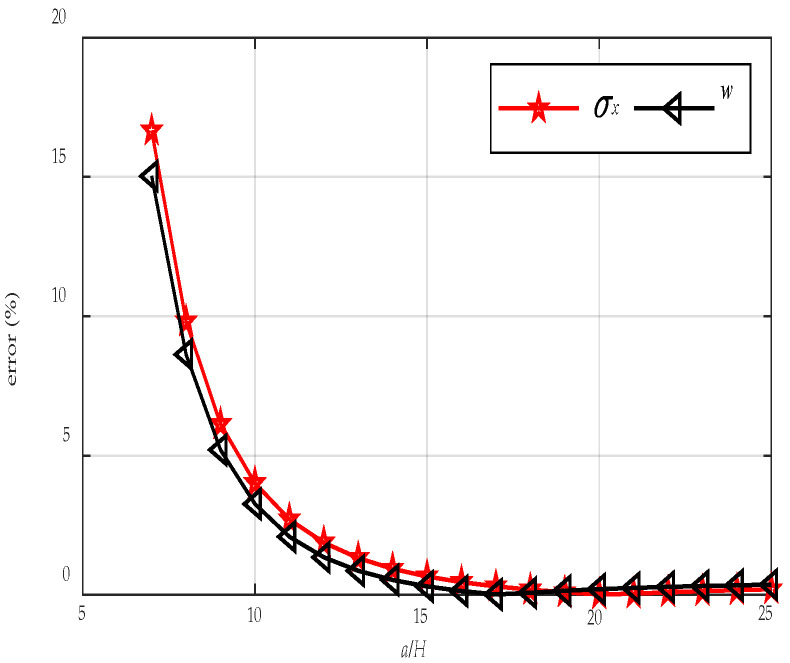
Variations in the maximum relative errors in *σ_x_* and *w* with respect to *a*/*H*.

In addition, the present solution is compared with the analytical solution based on the Kirchhoff plate theory [29]. The research object is a simply supported plate with two facial layers sandwiching a thin interlayer under the PL condition. The geometric and material parameters are *h*_1_ = *h*_3_ = 50 mm, *h*_2_ = 0.4 mm, *E*_1_ = *E*_3_ = 55,000 MPa and *E*_2_ = 6.12 MPa. Figure 5 illustrates the variations in the maximum relative errors of *σ_x_* and *w* with respect to length-to-thickness ratio *a*/*H*. It can be observed from Figure 5 that when *a*/*H* is small, the discrepancies between the two results are substantial. At *a*/*H* = 7, the errors reach 16.66% and 15.03%. For 7 < *a*/*H* < 15, the errors decrease markedly with increasing *a*/*H*, while the rate of reduction gradually diminishes. When *a*/*H* exceeds 15, the errors become negligible. This is because the Kirchhoff theory neglects the transverse shear deformation of the plate, which becomes significant when the plate is relatively thick, and this is also consistent with the viewpoint reported in the literature [30].

### 4.2. Effect of Wood Specie

**Figure 6:**
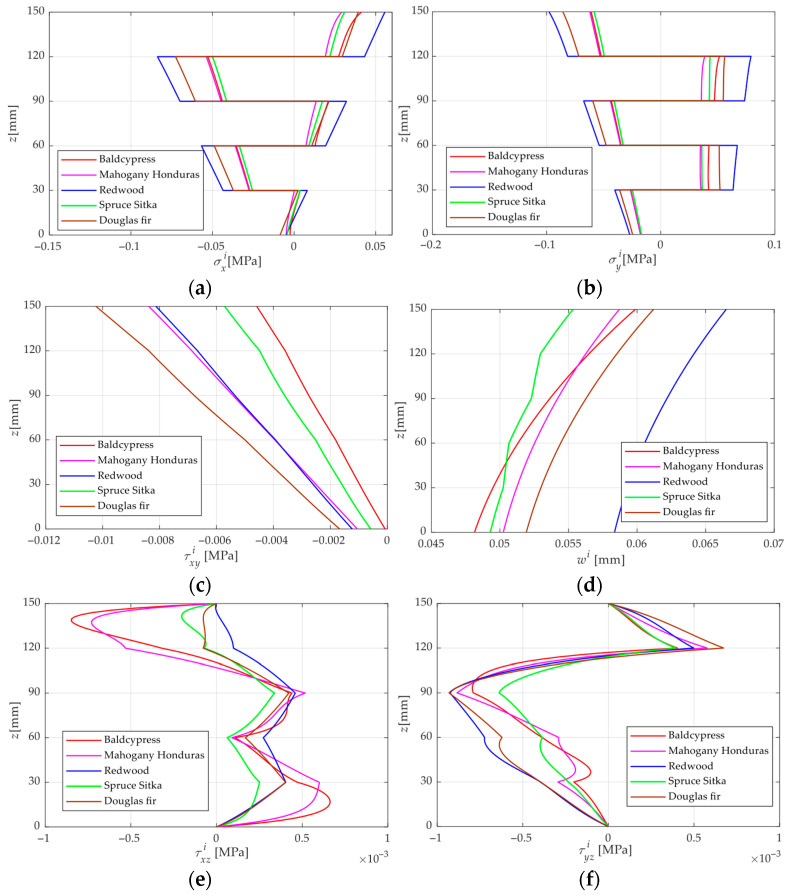

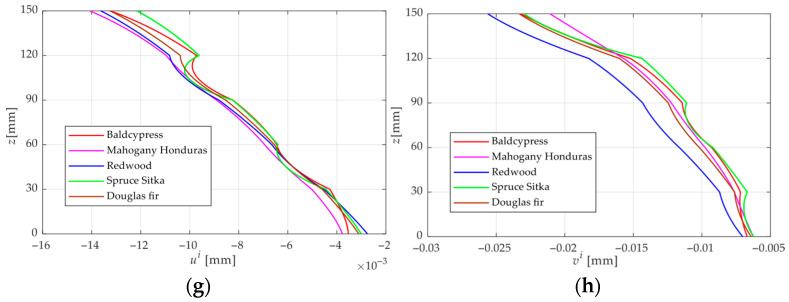
*z*-direction distributions of stresses and displacements of CLT plates made of five different wood species. (**a**) $\sigma_{x}^{i}$, (**b**) $\sigma_{y}^{i}$, (**c**) $\tau_{xy}^{i}$, (**d**) *w^i^*, (**e**) $\tau_{xz}^{i}$, (**f**) $\tau_{yz}^{i}$, (**g**) *u^i^*, (**h**) *v^i^*. Note: the values of $\sigma_{x}^{i}$, $\sigma_{y}^{i}$, *w* are located at the point of *x* = 0.5 *L*, *y* = 0.5 *W*, $\tau_{xy}^{i}$, $\tau_{xz}^{i}$, $\tau_{yz}^{i}$ at *x* = 0.25 *L*, *y* = 0.25 *W*, *u* at *x* = 0, *y* = 0.5 *W* and *v* at *x* = 0.5 *L*, *y* = 0.

Figure 6 illustrates the stress and displacement distributions along the thickness of the CLT plates made of five different woods under the PM condition with *M_t_* = 0.205, *M_b_* = 0.062. The following can be observed from Figure 6: (i) The stress and displacement distributions of the five different CLT plates exhibit generally similar patterns. $\sigma_{x}^{i}$ and $\sigma_{y}^{i}$ vary discontinuously through the thickness and show a zigzag distribution. Unlike the normal stress, $\tau_{xz}^{i}$ and $\tau_{yz}^{i}$ also exhibit a zigzag pattern but remain continuous along the thickness. Overall, $\tau_{xy}^{i}$, $u^{i}$, $v^{i}$ and $w^{i}$ decrease monotonically from the upper surface to the lower surface, while $u^{i}$ and $v^{i}$ exhibit pronounced nonlinear distributions in some layers, indicating that the cross-section of the CLT plate under PM is no longer planar. (ii) Higher stress and displacement levels are observed near the top surface, caused by the moisture gradient being mainly concentrated on the high-moisture side and dominating the through-thickness deformation. (iii) Owing to the anisotropy of wood, moisture-induced stresses differ substantially between odd- and even-numbered laminae, with adjacent layers usually having opposing stress orientations. (iv) The magnitudes of moisture-induced stresses and displacements rise with the elastic modulus *E*, shear modulus *G*, and the ratio *β_L_*/*β_T_*. Within the layers, Redwood exhibits the highest stress in *σ_x_*, indicating that the stress is predominantly governed by the elastic modulus. Meanwhile, the pronounced discontinuities of *σ_x_* across the interfaces reveal that the variation in elastic modulus between adjacent layers governs the stress redistribution. Furthermore, the moisture expansion coefficient and its interlayer discrepancy also contribute to the development of *σ_x_*. The deflection *w* is mainly governed by the moisture expansion coefficient and its difference between adjacent layers. The above analyses of commonly used wood species for CLT plates can provide guidance for the material selection of CLT plates in practical engineering applications under moisture service environments.

### 4.3. Effect of Surface MC Difference

**Figure 7:**
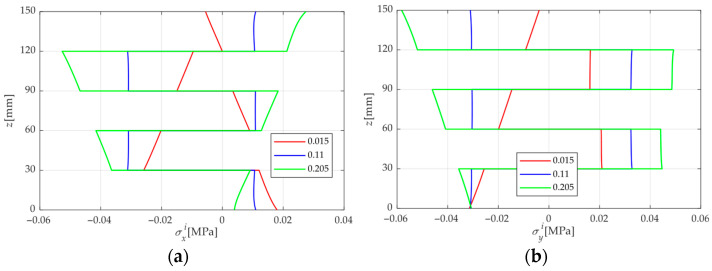

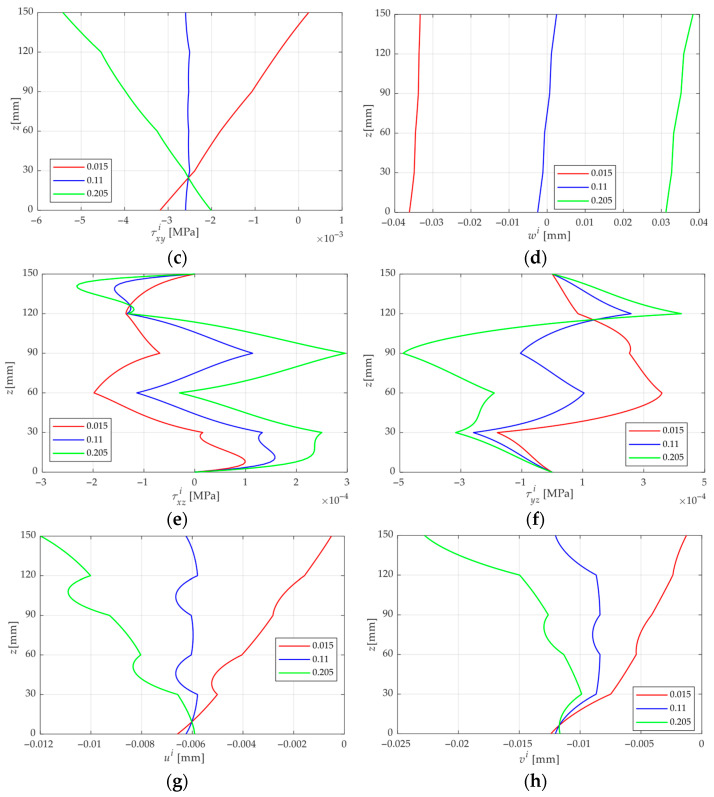
Distributions of stress and displacement along the thickness of the CLT plate under three different MCs. (**a**) $\sigma_{x}^{i}$, (**b**) $\sigma_{y}^{i}$, (**c**) $\tau_{xy}^{i}$, (**d**) *w^i^*, (**e**) $\tau_{xz}^{i}$, (**f**) $\tau_{yz}^{i}$, (**g**) *u^i^*, (**h**) *v^i^*. Note: the values of $\sigma_{x}^{i}$, $\sigma_{y}^{i}$, *w* are located at the point of *x* = 0.5 *L*, *y* = 0.5 *W*, $\tau_{xy}^{i}$, $\tau_{xz}^{i}$, $\tau_{yz}^{i}$ at *x* = 0.25 *L*, *y* = 0.25 *W*, *u* at *x* = 0, *y* = 0.5 *W* and *v* at *x* = 0.5 *L*, *y* = 0.

Figure 7 illustrates the effect of top and bottom surface MC differences on the mechanical response of the Spruce Sitka CLT plate, with *M_b_* = 0.11, *M_t_* = 0.205, 0.11, and 0.015, respectively. It can be found from Figure 7 that when the surface MC is identical, $\sigma_{x}^{i}$, $\sigma_{y}^{i}$, $\tau_{xy}^{i}$, $u^{i}$ and $v^{i}$ are symmetric about the mid-plane, while $\tau_{xz}^{i}$, $\tau_{yz}^{i}$ and $w^{i}$ are anti-symmetric. This is completely opposite to the symmetry observed under the PL condition in Section 4.1. This is because the CLT plate is materially symmetric with respect to the mid-plane. Under the PM condition, the CLT plate bends toward one side. In this section, the swelling is symmetric across the plate’s upper and lower faces, and the moisture-induced responses also obey the principle of superposition. Specifically, the stresses and displacements at *M_t_* = 0.11 are approximately equal to the average of those obtained under *M_t_* = 0.015 and *M_t_* = 0.205. However, slight discrepancies occur due to the MDP of wood under different MCs. The analysis of surface MC differences in CLT can be used for the design of CLT panels employed in the exterior walls and roofing of timber buildings.

## 5. Conclusions

Analytical solutions grounded in 3D elasticity theory are formulated to examine the flexural response of CLT plates under combined load and moisture effects. The main findings of this study are summarized below:

1. The FE results tend to agree with the present analytical solution as the FE mesh becomes finer. When the number of finite element elements reaches 6.25 × 10^9^, the errors in the typical normal stress, shear stress, and deflection compared with the present solution are 1.59%, 0.0901%, and 1.02%, respectively. In addition, the FE results exhibit relatively large discrepancies near the surfaces. The analytical solution provides significantly higher computational efficiency in predicting the LM-coupled response of CLT plates than the FE simulations, as the latter require increasingly longer computation times when the mesh is refined.
2. The Kirchhoff plate solution agrees well with the present solution when the length-to-thickness ratio of the CLT plate is large; however, the discrepancy increases markedly as the plate becomes thicker. When *a*/*H* = 7, the errors of normal stress, and deflection reach 16.66% and 15.03%. This is due to the transverse shear deformation of the plate being neglected in the Kirchhoff theory.
3. For the mechanical analysis of CLT plates under LM conditions, it is found that the conventional superposition principle is no longer applicable, leading to errors of up to 18.2%. This study proposes a modified superposition principle that accounts for the effect of MDP under PL conditions in addition to the traditional superposition approach.
4. When the MC at the top surface of the CLT plate exceeds that at the bottom, stress and displacement generally occur in opposing directions. This is because the external load tends to bend the plate downward, whereas the moisture-induced expansion on the upper surface drives an upward deformation. Consequently, under LM coupling, these opposing flexural effects partially compensate for each other, thus reducing the overall stress and deformation of the CLT plate.
5. For a CLT plate under PL conditions, the stresses and displacements exhibit clear symmetric or antisymmetric distributions with respect to the mid-plane of the plate. However, such symmetry occurs only when the CLT is under uniform moisture content, and in this case, the symmetry is exactly opposite to that observed under PL conditions.
6. Through the analysis of mechanical behaviors of different wood species under the PM condition, it can be concluded that the primary factors governing the moisture-induced stress and displacement responses are the elastic modulus, shear modulus, and the relative magnitude of the moisture expansion coefficient. Owing to the pronounced elastic modulus anisotropy between the longitudinal and tangential directions, moisture-induced stresses differ markedly between odd and even laminae, with adjacent layers generally exhibiting opposite stress directions.

## Figures and Tables

**Table 1:** Basic material parameters of five types of woods.

Material Property	Bald Cypress	Mahogany Honduras	Redwood	Spruce Sitka	Douglas Fir
*E_L_* (12%)	10,890	11,330	10,120	11,880	13,297.7
*E_L_* (green)	8910	10,120	8910	9350	10,717.5
*E_T_*/*E_L_*	0.039	0.064	0.089	0.043	0.050
*E_R_*/*E_L_*	0.084	0.107	0.087	0.078	0.068
*G_LR_*/*E_L_*	0.063	0.066	0.066	0.064	0.064
*G_LT_*/*E_L_*	0.054	0.086	0.077	0.061	0.078
*G_RT_*/*E_L_*	0.007	0.028	0.011	0.003	0.007
*μ_LR_*	0.338	0.314	0.36	0.372	0.292
*μ_LT_*	0.326	0.533	0.346	0.467	0.449
*μ_TL_*	0.013	0.034	0.031	0.025	0.029
*μ_TR_*	0.356	0.326	0.4	0.245	0.374
*λ_L_*	1 × 10^−7^	2.5 × 10^−7^	5 × 10^−7^	1.8 × 10^−7^	5 × 10^−7^
*λ_T_*	6.2 × 10^−2^	4.2 × 10^−2^	4 × 10^−8^	7 × 10^−8^	5 × 10^−8^
*λ_R_*	3.8 × 10^−2^	3 × 10^−2^	4 × 10^−8^	7 × 10^−8^	5 × 10^−8^
*β_L_*	2 × 10^−5^	2 × 10^−5^	1 × 10^−5^	2 × 10^−5^	2 × 10^−5^
*β_T_*	6.2 × 10^−4^	4.2 × 10^−4^	5 × 10^−4^	5 × 10^−4^	5.5 × 10^−4^
*β_R_*	3.8 × 10^−4^	3 × 10^−4^	2.4 × 10^−4^	3 × 10^−4^	2.8 × 10^−4^
*M_p_*	0.25	0.25	0.21	0.27	0.24

Note: the units of *E* and *λ* are [MPa] and [m^2^/s], respectively. The values in parentheses, 12% and green, refer to the elastic modulus at 12% MC and in the green wood state, respectively.

**Table 2:** Comparison of deflection among the present analytical model, the FE simulations and the experimental data.

Solution	Present	FE	Experiment Repeated Tests			
			1	2	3	Average
3-layer	62.49	63.24	59.5	69.47	75.12	68.03
5-layer	83.77	84.69	85.76	87.19	104	92.32

## Data Availability

The original contributions presented in this study are included in the article. Further inquiries can be directed to the corresponding author.

## Nomenclature

CLT, cross-laminated timber; MC, moisture content; LM, load-moisture; MDP, moisture-dependent property; 3D, three-dimensional; FE, finite element; PL, pure load; PM, pure moisture.

## Appendix A

The matrices $S_{mn}^{i}$ and $M_{mn}^{i}(z)$ in Equation (9) are given as follows:

$$S_{mn}^{i}=\left[\begin{array}{cccccccc} 0 & -\frac{1}{\lambda_{z}^{i}} & 0 & 0 & 0 & 0 & 0 & 0\\ -\lambda_{z}^{i}(a_{m}^{2}+b_{n}^{2}) & 0 & 0 & 0 & 0 & 0 & 0 & 0\\ 0 & 0 & 0 & 0 & -a_{m} & 0 & \frac{1}{c_{55}^{i}} & 0\\ 0 & 0 & 0 & 0 & -b_{n} & 0 & 0 & \frac{1}{c_{44}^{i}}\\ 0 & 0 & \frac{a_{m}c_{13}^{i}}{c_{33}^{i}} & \frac{b_{n}c_{23}^{i}}{c_{33}^{i}} & 0 & \frac{1}{c_{33}^{i}} & 0 & 0\\ 0 & 0 & 0 & 0 & 0 & 0 & a_{m} & b_{n}\\ 0 & 0 & f_{1}^{i}a_{m}^{2}+c_{66}^{i}b_{n}^{2} & (f_{2}^{i}+c_{66}^{i})a_{m}b_{n} & 0 & -\frac{a_{m}c_{13}^{i}}{c_{33}^{i}} & 0 & 0\\ 0 & 0 & (f_{2}^{i}+c_{66}^{i})a_{m}b_{n} & f_{3}^{i}b_{n}^{2}+c_{66}^{i}a_{m}^{2} & 0 & -\frac{b_{n}c_{23}^{i}}{c_{33}^{i}} & 0 & 0 \end{array}\right],$$

(A1) $$M_{mn}^{i}(z)=\left[\begin{array}{c} 0\\ 0\\ 0\\ 0\\ \frac{c_{13}^{i}\beta_{x}^{i}+c_{23}^{i}\beta_{y}^{i}+c_{33}^{i}\beta_{z}^{i}}{c_{33}^{i}}M_{mn}^{i}\\ 0\\ a_{m}(f_{1}^{i}\beta_{x}^{i}+f_{2}^{i}\beta_{y}^{i})M_{mn}^{i}\\ b_{n}(f_{2}^{i}\beta_{x}^{i}+f_{3}^{i}\beta_{y}^{i})M_{mn}^{i} \end{array}\right],\;\left[\begin{array}{c} f_{1}^{i}\\ f_{2}^{i}\\ f_{3}^{i} \end{array}\right]=\frac{1}{c_{33}^{i}}\left[\begin{array}{c} c_{11}^{i}c_{33}^{i}-{\left(c_{13}^{i}\right)}^{2}\\ c_{12}^{i}c_{33}^{i}-c_{13}^{i}c_{23}^{i}\\ c_{22}^{i}c_{33}^{i}-{\left(c_{23}^{i}\right)}^{2} \end{array}\right].$$
